# Long-term prognostic factors and outcomes in mitochondrial encephalomyopathy with lactic acidosis and stroke-like episodes: a clinical and biochemical marker analysis

**DOI:** 10.3389/fneur.2024.1491283

**Published:** 2024-12-04

**Authors:** Rui Gao, Lihua Gu, Wenchao Zuo, Pan Wang

**Affiliations:** Department of Neurology, Tianjin Huanhu Hospital, Nankai University, Tianjin, China

**Keywords:** MELAS, prognosis, clinical markers, biochemical markers, risk factor

## Abstract

**Background:**

MELAS (Mitochondrial Encephalomyopathy, Lactic Acidosis, and Stroke-like episodes) is a common subtype of mitochondrial encephalomyopathy. However, few studies have explored the relationship between biochemical markers and prognosis. This study aimed to explore the relationship between clinical and biochemical markers and prognosis of patients with MELAS.

**Methods:**

This was a retrospective single-center study. A total of 39 MELAS patients were followed for an average of 7.3 ± 4.7 (range 1–21 years). All patients underwent detailed demographic registration, neurological examinations, biochemical and mitochondrial DNA analyses, muscle biopsy. Throughout the follow-up period, the modified Rankin Scale (mRS) scores, recurrent strokes rates, and mortality were tracked.

**Results:**

All patients initially presented with stroke-like episodes. Of the 39 subjects who were followed, 8 died, primarily due to acute stroke-like episodes and status epilepticus. Univariate analysis showed a higher risk of mortality in patients with severe lactate elevation compared to those with normal and mildly elevated levels (OR = 5.714, 95% CI 1.086–30.071, *p* = 0.040). While the absence of anemia was associated with a lower risk of death compared to those with anemia (OR = 0.175, 95% CI 0.033–0.921, *p* = 0.040). In multivariate analysis, severe lactate elevation (OR = 7.279, 95% CI 1.102–48.086, *p* = 0.039) and anemia (OR = 0.137, 95% CI 0.021–0.908, *p* = 0.039) were identified as independent predictors of mortality. MRS scores were categorized as follows: 41% of patients scored 0 to 2, 38.5% scored 3 to 5, and 20.5% had a score of 6 or had died. There was a positive correlation between lactic acid levels and MRS scores (r = 0.460, *p* = 0.003). In contrast, hemoglobin levels were negatively correlated with MRS scores (r = −0.375, *p* = 0.015). Furthermore, a positive correlation was observed between MRS scores and the frequency of stroke-like episodes (r = 0.280, *p* = 0.042).

**Conclusion:**

Our study found that the majority of patients with MELAS had poor clinical outcomes. Anemia and significantly increased lactate levels were identified as indicators of poor prognosis in MELAS. Early intervention may lead to improvements in clinical outcomes.

## 1 Introduction

Mitochondrial Encephalomyopathy, Lactic Acidosis, and Stroke-like Episodes (MELAS) is a prevalent maternally inherited mitochondrial disorder. It is a multi-system disorder with a wide range of manifestations, characterized by recurrent stroke-like episodes, progressive intellectual decline, and dementia (1). MELAS is associated with high morbidity and mortality rates. Among patients with the m.3243A > G mutation, earlier central nervous system involvement is often associated with a more severe progression of the disease (2). However, research on the risk factors for mortality in MELAS is limited, primarily focusing on clinical symptoms, with even fewer studies investigating biochemical indicators. For instance, an 8-year follow-up study on clinical indicators and their prognostic implications in a Chinese cohort (3), a 5-year follow-up study in a Japanese cohort (4), and a study examining baseline clinical characteristics, mutation load in urine and blood, and major adverse events in a French cohort (5) have been conducted. We conducted a retrospective follow-up study with a follow-up period of up to 21 years on a cohort of MELAS patients to examine the relationships between clinical and biochemical parameters, cerebrospinal fluid lactate levels, and prognosis.

## 2 Methods

### 2.1 Subjects

In this retrospective, single-center study, the cohort included symptomatic MELAS patients treated at Tianjin Huanhu Hospital between January 2003 and January 2023. All participants met the diagnostic criteria established in previously published studies (6–8). All patients underwent full-length mitochondrial DNA (mtDNA) sequencing and/or relevant nuclear gene testing, as well as muscle biopsy.

### 2.2 Clinical and biochemical data collection

The onset of MELAS was defined by the presence of initial symptoms or events, such as seizures, stroke-like episodes, or severe headaches, which were associated with neuroimaging abnormalities (4). Patient characteristics, including gender, age at onset, prodromal symptoms, and clinical manifestations, were recorded. Biochemical data collected at the onset of the disease included cerebrospinal fluid lactate, venous blood lactate, creatine kinase (CK), CK-MB, creatinine, cholesterol, hemoglobin, triglycerides, uric acid, fasting blood glucose, and glycated hemoglobin. Mild hyperlactatemia was defined as a blood lactate level greater than the upper limit of the normal range (>2 mmol/L), while severe hyperlactatemia was defined as a blood lactate level greater than twice the upper limit of the normal range (>4 mmol/L). Similarly, mild elevation in cerebrospinal fluid (CSF) lactate was defined as a level exceeding the upper normal limit (>2.2 mmol/L), and severe elevation was defined as a level exceeding two times the upper normal limit (>4.4 mmol/L). The levels of CK, hemoglobin, creatinine, and uric acid were adjusted according to reference values specific to age and gender. All biochemical markers were collected during fasting and resting states to minimize variability. Cerebrospinal fluid was obtained via lumbar puncture, performed under sterile conditions.

### 2.3 Cohort follow-up

The follow-up period concluded in January 2024. The follow-up duration ranged from 1 to 21 years, with medical histories obtained through face-to-face visits or telephone interviews. Using the mRS, patients’ disability status was assessed, and mortality data were collected (9). Importantly, none of the patients were lost to follow-up throughout the study period.

### 2.4 Statistical analysis

The Mann–Whitney U test was utilized to detect differences between continuous variables, while Fisher’s exact test was used for categorical variables. The relationships between mRS scores, lactate levels, hemoglobin levels, and age were assessed using Kendall’s tau-b correlation coefficient. Univariate and multivariate analyses were performed using a binary logistic regression model. To evaluate the potential influence of follow-up duration on the results, a sensitivity analysis was conducted. This included follow-up time categorized into intervals (0–4 years, 4–8 years, 8–12 years, 12–16 years, 16–20 years, and over 20 years) in the multivariable logistic regression analysis. Data analysis was performed using SPSS version 29.0. A *p*-value of less than 0.05 was regarded as statistically significant.

## 3 Results

### 3.1 Demographic and pathological findings of MELAS in the cohort study

A total of 39 patients were enrolled in the study. Of these, approximately 64% of patients were male and 36% were female. The mean age at the onset of the disease was 33.5 ± 10.2 years. A positive family history was noted in 74.4% of the patients. Pathogenic mutations of mtDNA were identified in 39 patients, including m.3243A > G (*n* = 30) and m.3136A > G (*n* = 1). The remaining 8 patients met the Bernier criteria (7), and were definitively diagnosed through muscle biopsies. Muscle biopsies were performed on all 39 patients. Ragged-red fibers (RRF) were observed in 29 patients (74.4%), and SDH strongly reactive blood vessels (SSV) were observed in 28 patients (71.8%). The mean time interval from the first episode to the last visit at our institute or death was 7.5 ± 5.2 years. The average number of stroke-like episodes during the follow-up period was 3.2 ± 1.5 (ranging from 1 to 6). Among the 36 patients who were followed up, 8 died. The mean age of the patients who died was 37.8 ± 12.1 years. The time from onset to death was 4.1 ± 2.5 years. Six patients died from stroke-like episodes, with status epilepticus being the clinical manifestation prior to death. The remaining deaths were attributed to cardiac insufficiency (1) and pulmonary infection (1; Table 1).

**Table 1:** Demographic and pathological characteristics in MELAS cases.

Demographic and pathological findings	Total^1^
Patient (male/female)	39 (25/14)
Age of onset, years	33.5 ± 10.2 (14–55)
Positive family history	29 (74.4%)
Muscle biopsy examination	39
RRF	29 (74.4%)
SSV	28 (71.8%)
A3243G mutation positive	30 (76.9%)
A3136G mutation positive	1 (0.03%)
Mutation not found	8 (20.5%)
Duration of follow up	7.3 ± 4.7 (1–21)
Number of stroke-like episodes	3.2 ± 1.5 (1–6)
Mrs (0–2)	16 (41)
Mrs (3–5)	15 (38.5)
Mrs6	8 (20.5)
Age of death, years	37.8 ± 12.1 (22–58)
Time from onset to death	4.1 ± 2.5 (1–9)

1 Absolute number (%) or Mean ± standard deviation (Minimum to maximum).

### 3.2 Clinical manifestations before onset and onset

All the patients in our study exhibited stroke-like episodes. Prior to the onset of the condition, the most frequently observed symptoms included hearing loss, general fatigue, and short stature, which were reported by the patients or noted during assessments. At the onset of the stroke-like episodes, the predominant symptoms observed were headache and seizures. Additional clinical symptoms observed both before and at onset are listed in Table 2.

**Table 2:** Clinical symptoms before and at disease onset.

Clinical manifestations	Total^1^
Clinical manifestations before onset	
Hearing loss	27 (69.2%)
diabetes mellitus	19 (48.7%)
Seizure	8 (20.5%)
Short stature	25 (64.1%)
General fatigue	27 (69.2%)
Cardiac dysfunction	18 (46.2%)
Symptoms at onset	
Headache	31 (79.5%)
Seizure	28 (71.8%)
Status epilepticus	18 (46.2%)
Cortical blindness	18 (46.2%)
Focal weakness	12 (30.8%)
Ataxia	10 (25.6%)
Dysphasia	22 (56.4%)
Sensory disturbance	8 (20.5%)
Cognitive disorder	26 (66.7%)
Psychonosema	23 (59%)
peripheral neuropathy	17 (43.6%)
Fever	9 (23.1%)
Thyroid diseases	7 (17.9%)
Ileus	3 (7.7%)

1 Absolute number (%).

### 3.3 Cerebrospinal fluid and blood parameters

In our study, the elevation of lactate levels in blood and cerebrospinal fluid (CSF) was observed as follows: mild elevations in blood lactate levels were seen in 69.2% of patients, while severe elevations were noted in 30.8% of patients. For CSF lactate testing, mildly elevated levels were observed in 94.9% of patients, and severe elevations were noted in 38.5%. Blood hemoglobin tests indicated anemia, defined as hemoglobin levels below the normal range, in 30.8% of patients. A small proportion of patients exhibited abnormally elevated levels of blood CK, CK-MB, creatinine, cholesterol, triglycerides, and uric acid. The proportion of patients with abnormally elevated fasting blood glucose levels showed a roughly equal distribution compared to those without such elevations, and a similar pattern was observed for glycated hemoglobin levels (Table 3).

**Table 3:** Biochemical markers in cerebrospinal fluid and blood.

Assay index	Subset	Total^1^
Blood lactate (mmol/L)		
Mildly increased	≤2	12 (30.8%)
	>2	27 (69.2%)
Severely increased	≤4	27 (69.2%)
	>4	12 (30.8%)
Cerebrospinal fluid lactic acid (mmol/L)		
Mildly increased	≤2.2	2 (5.1%)
	>2.2	37 (94.9%)
Severely increased	≤4.4	24 (61.5%)
	>4.4	15 (38.5%)
CK (UL)	≤310/200^a^	28 (71.8%)
	>310/200^a^	11 (28.2%)
Ckmb (UL)	≤24	28 (71.8%)
	>24	11 (28.2%)
Creatinine (μmol/L)	≤97/73^b^	36 (92.3%)
	>97/73^b^	3 (7.7%)
Hemoglobin (g/L)	≤130/115^c^	12 (30.8%)
	>130/115^c^	27 (69.2%)
Triglyceride (mmol/L)	≤1.7	27 (69.2%)
	>1.7	12 (30.8%)
Cholesterol (mmol/L)	≤5.18	33 (84.6%)
	>5.18	6 (15.4%)
Fasting blood glucose (mmol/L)	≤6.1	22 (56.4%)
	>6.1	17 (43.6%)
Uric acid (μmol/L)	≤420/350^d^	33 (84.6%)
	>420/350^d^	6 (15.4%)
Glycated hemoglobin (%)	≤6	18 (46.2%)
	>6	21 (53.8%)

These values represent the patient demographics and hemoglobin levels observed in our study.

1 Absolutenumber (%).

a Male: 310, Female: 200.

b Male: 97, Female: 73.

c Male: 130, Female: 115; all hemoglobin values were below the upper limit of normal.

d Male: 420, Female: 350.

### 3.4 Risk factors for mortality

In the univariate analysis, patients with severe hyperlactatemia had a higher risk of mortality compared to those with normal and mildly elevated lactate levels (OR = 5.714, 95% CI 1.086–30.071, *p* = 0.040). Patients without anemia had a lower risk of death compared to those with anemia (OR = 0.175, 95% CI 0.033–0.921, *p* = 0.040). Multifactorial analysis, incorporating single positive indicators along with gender and age, showed that patients with severe hyperlactatemia had a higher risk of mortality compared to those with normal and mildly elevated lactate levels (OR = 7.279, 95% CI 1.102–48.086, *p* = 0.039). Additionally, patients without anemia had a lower risk of death compared to those with anemia (OR = 0.137, 95% CI 0.021–0.908, *p* = 0.039; Supplementary Table S1). We further evaluated the impact of follow-up duration on the results, and the sensitivity analysis did not alter the findings of the primary analysis (Supplementary Table S2).

### 3.5 mRS scores

**Figure 1:**
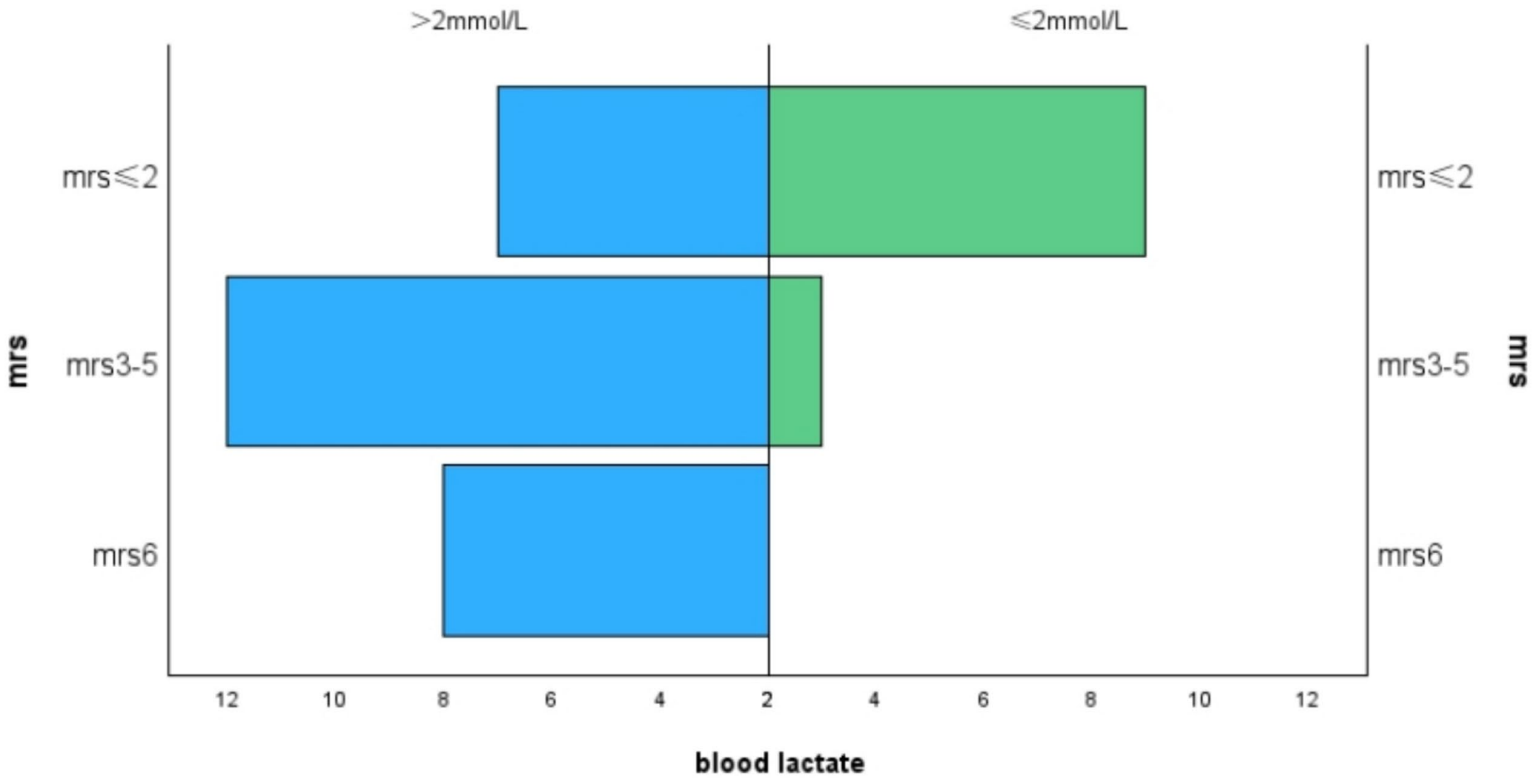
Correlation between MRS and blood lactate levels.

**Figure 2:**
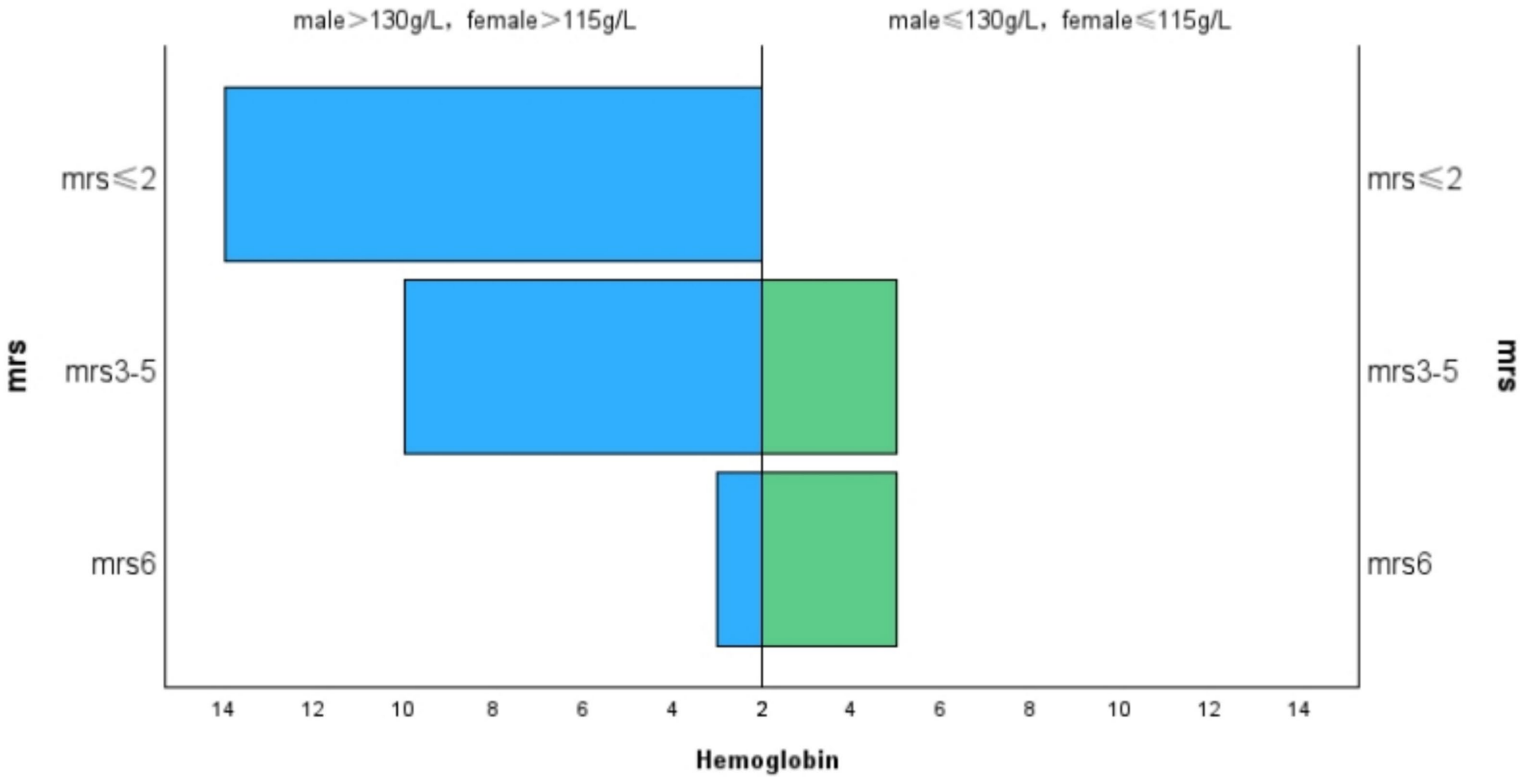
Correlation between MRS and hemoglobin.

The average mRS score of the patients in our study was 3.18 ± 1.57. We divided the MRS scores into three levels:41% of patients had an mRS score of 0–2,38.5% had a score of 3–5, and 20.5% either died or had an mRS score of 6. Lactate levels exhibited a positive correlation with mRS scores (r = 0.460, *p* = 0.003, Figure 1), and hemoglobin levels were negatively correlated with mRS scores (r = −0.375, *p* = 0.015, Figure 2). Additionally, we discovered a positive correlation between mRS scores and the frequency of stroke-like episodes (r = 0.280, *p* = 0.042). However, we did not find a correlation between mRS scores and the age of onset (r = −0.114, *p* = 0.374).

## 4 Discussion

MELAS syndrome is characterized by high mortality and morbidity. Previous research demonstrated that the mortality rate was higher in MELAS patients who had focal seizures or stroke-like episodes (10). These findings suggest that identifying risk factors for mortality or disability may help predict the clinical course of the disease and facilitate early clinical intervention. We conducted a multivariate analysis to identify clinical and biochemical factors associated with poor prognosis in MELAS.

The average mRS score of the patients in this study was 3.18 ± 1.57, which is consistent with previous studies reporting an mRS score of 3.3 ± 1.8 (3), indicating a poor prognosis for the majority of patients. Among the 8 deceased MELAS patients, the average age at death was 37.8 ± 12.1 years, which was higher than the 23.3 ± 11.9 years reported in a study involving Chinese patients (3), but similar to the 34.5 ± 19 years observed in another study of American patients (10).

The main causes of death in this study were stroke-like episodes and status epilepticus, which aligns with findings from previous research. Zhang et al. reported that most deaths among MELAS patients resulted from stroke-like episodes, which frequently presented with clinical manifestations such as status epilepticus and metabolic acidosis (3). Moreover, Fayssoil et al. observed that the majority of deaths among their MELAS patients were attributed to stroke-like episodes and status epilepticus, while other causes included heart failure and bowel obstruction (5). These studies underscore the critical importance of early identification and treatment of stroke-like episodes and status epilepticus.

One patient in our cohort succumbed to heart failure. Heart failuer has been reported as the cause of death for 71% of adult patients with the m.3243A > G mutation (11). Given the high rate of left ventricular involvement, it is recommended that patients with MELAS syndrome undergo regular cardiac examinations (12). One patient in our cohort died from a lung infection, which aligns with prior studies reporting mortality due to complications such as dyspnea and pulmonary edema (12). Kaufmann et al. reported that intestinal obstruction is another significant cause of mortality among MELAS patients (10), highlighting the need for comprehensive care that includes gastrointestinal assessment. These observations underscore the necessity of addressing not only neurological symptoms in MELAS patients but also monitoring for potential damage to vital organs, including the heart, lungs, and gastrointestinal system.

According to a prognostic study conducted by Zhang et al. on Chinese MELAS patients, a negative correlation was observed between mRS scores and age of onset, indicating that an earlier onset of the disease is associated with more severe outcomes. Additionally, stroke-like episodes served as independent predictors of death (3). Conversely, a clinical investigation in Taiwan on MELAS patients carrying the m.3243A > G mutation identified seizures and status epilepticus as the most significant predictors of unfavorable prognosis, while age of onset and visceral organ damage did not significantly impact outcomes (12). In a study of 43 adult patients in France, Fayssoil et al. identified a urine mutation load of ≥45%, along with left ventricular hypertrophy and seizures, as significant factors associated with hospitalization or death (5). In this study, we found a positive correlation between mRS scores and the frequency of stroke like episodes. This finding suggests that increased frequency of central nervous system involvement may correlate with more severe clinical manifestations in MELAS patients.

The multivariate analysis indicated that significant elevations in lactate levels were associated with an increased risk of death. Elevated lactate levels correlated with higher mRS scores, reflecting greater disability. The majority of patients with MELAS syndrome exhibited elevated lactate levels in both blood and cerebrospinal fluid, as supported by previous studies (13). Mitochondrial dysfunction led to increased anaerobic glycolysis in muscle tissues, resulting in elevated blood lactate levels due to insufficient oxidative phosphorylation (14). Previous studies have identified serum lactate as an independent risk predictor for stroke-like episodes in carriers of the m.3243A > G mutation, suggesting a potential association with poor prognosis (15). Thus far, the most significant biomarker indicating the severity of MELAS disease is the lactate level measured in the ventricles through brain magnetic resonance spectroscopy (16), suggesting that elevated CSF lactate correlates with worsening disease severity (10). In individuals with the m.3243A > G mutation, the levels of heteroplasmy in skeletal muscle and blood have been positively associated with disease severity (17). Given the small sample size of this study, further investigation with larger cohorts is needed to ascertain whether severely elevated blood lactate consistently correlates with poor prognosis and mortality in MELAS patients.

Hematological abnormalities, including anemia and various forms of cytopenia, have been reported in approximately 10 to 30% of patients with mitochondrial diseases (18). Defects in the mitochondrial respiratory chain impair the differentiation capacity of hematopoietic stem cells, resulting in cytopenias (18, 19). Our study found that 30.8% of patients exhibited anemia, a slightly higher proportion than expected, potentially influenced by patient selection criteria, though dietary or other factors affecting hemoglobin levels could not be ruled out. Research on POLG-related mitochondrial diseases has also demonstrated that anemia is significantly associated with worse prognosis (20).

Currently, several small cohort studies have investigated potential treatments for MELAS. A study involving two MELAS patients indicated that taurine administration resulted in no further seizures or stroke-like episodes, accompanied by a reduction in serum lactate and pyruvate levels (21). An investigation demonstrated that after 48 weeks of sodium pyruvate treatment in patients with mitochondrial disease, blood lactate concentration and lateral ventricular lactate levels significantly declined from baseline, showing a clear trend of improvement in the Newcastle Mitochondrial Disease Adult Scale (NMDAS) partial scores (22). Arginine administration was found to lower blood lactate concentrations in MELAS patients (13) and to reduce the frequency of stroke-like episode recurrences (23). Recent studies have indicated that the mammalian target of rapamycin inhibitor rapamycin promotes erythroid differentiation in pluripotent stem cells and significantly improves the anemic phenotype in patients with Mitochondrial Leukoencephalopathy, Lactic Acidosis, and Stroke-like episodes (24).

This study has several limitations. First, as a retrospective cohort study, it was inherently vulnerable to recall bias. Secondly, selection bias may have been introduced, as younger patients could have sought treatment at pediatric hospitals, despite our institution being a specialized neurology center. Additionally,. our multivariate analysis primarily focused on clinical variables and biochemical markers, without thoroughly investigating the relationships between clinical phenotypes, mtDNA heteroplasmy, or emerging biomarkers such as fibroblast growth factor 21 (FGF-21) and growth differentiation factor 15 (GDF-15) (25), both of which have been linked to mitochondrial disease severity (26). Future studies should investigate these biomarkers, along with genetic mutations, to gain a more comprehensive understanding of their prognostic significance. Although standard protocols for biochemical data collection were followed, the venous lactate levels might have been artificially elevated as a result of patient restlessness during the sampling process (27).

In conclusion, our study demonstrated that severe elevations in venous lactate levels and the presence of anemia are associated with increased mortality and adverse outcomes in patients with MELAS. Early identification of these indicators could aid in assessing patient prognosis and facilitate graded management, potentially leading to improved outcomes.

## Data Availability

The raw data supporting the conclusions of this article will be made available by the authors, without undue reservation.
