# Supplementary material for: Long-term prognostic factors and outcomes in mitochondrial encephalomyopathy with lactic acidosis and stroke-like episodes: a clinical and biochemical marker analysis
Source: Front Neurol. 2024 Dec 4;15:1491283. doi: 10.3389/fneur.2024.1491283 (PMC11652343; doi:10.3389/fneur.2024.1491283)
Supplement: Supplementary file 1 [file Table_1.docx]

Supplementary Table S1 Predictors of mortality

| Risk factor | Univariate analysis | | | Multivariate analysis^1^ | | |
| --- | --- | --- | --- | --- | --- | --- |
|  | OR | 95% CI | *p* value | OR | 95% CI | *P* value |
| gender | 3.500 | 0.694-17.693 | 0.129 |  |  |  |
| Age of onset,years | 0.998 | 0.924-1.078 | 0.959 |  |  |  |
| Positive family history | 1.043 | 0.174-6.257 | 0.963 |  |  |  |
| Clinical manifestations before onset |  |  |  |  |  |  |
| Hearing loss | 1.429 | 0.244-8.375 | 0.693 |  |  |  |
| diabetes mellitus | 1.067 | 0.225-5.049 | 0.935 |  |  |  |
| Seizure | 1.389 | 0.222-8.672 | 0.725 |  |  |  |
| Short stature | 1.895 | 0.327-10.968 | 0.476 |  |  |  |
| General fatigue | 1.429 | 0.244-8.375 | 0.693 |  |  |  |
| Cardiac dysfunction | 2.308 | 0.466-11.422 | 0.305 |  |  |  |
| Symptoms at onset |  |  |  |  |  |  |
| Headache | 2.042 | 0.213-19.529 | 0.536 |  |  |  |
| Seizure | 1.227 | 0.207-7.265 | 0.821 |  |  |  |
| Status epilepticus | 2.308 | 0.466-11.422 | 0.305 |  |  |  |
| Cortical blindness | 0.313 | 0.054-1.796 | 0.192 |  |  |  |
| Focal weakness | 0 | 0 | 0.999 |  |  |  |
| ataxia | 0 | 0 | 0.999 |  |  |  |
| Dysphasia | 1.373 | 0.278-6.775 | 0.697 |  |  |  |
| Sensory disturbance | 0.490 | 0.051-4.685 | 0.536 |  |  |  |
| cognitive disorder | 4.421 | 0.482-40.561 | 0.189 |  |  |  |
| Psychonosema | 0.330 | 0.066-1.650 | 0.177 |  |  |  |
| peripheral neuropathy | 2.639 | 0.531-13.116 | 0.236 |  |  |  |
| fever | 0 | 0 | 0.999 |  |  |  |
| Thyroid diseases | 0.595 | 0.61-5.801 | 0.655 |  |  |  |
| ileus | 10015944026 | 0 | 0.999 |  |  |  |
| blood lactate |  |  |  |  |  |  |
| mildly increased | 680199846.15 | 0 | 0.999 |  |  |  |
| Severely increased | 5.714 | 1.086-30.071 | 0.040* | 7.279 | 1.102-48.086 | 0.039* |
| Cerebrospinal fluid lactic acid |  |  |  |  |  |  |
| mildly increased | 445648176.97 | 0 | 0.999 |  |  |  |
| Severely increased | 3.500 | 0.694-17.639 | 0.129 |  |  |  |
| CK | 1.725 | 0.334-8.910 | 0.515 |  |  |  |
| Ckmb | 1.725 | 0.334-8.910 | 0.515 |  |  |  |
| Creatinine | 0 | 0 | 0.999 |  |  |  |
| Hemoglobin | 0.175 | 0.033-0.921 | 0.040* | 0.137 | 0.021-0.908 | 0.039* |
| Triglyceride | 2.875 | 0.579-14.275 | 0.196 |  |  |  |
| cholesterol | 2.250 | 0.332-15.256 | 0.406 |  |  |  |
| fasting blood glucose | 0.356 | 0.062-2.043 | 0.246 |  |  |  |
| uric acid | 0.743 | 0.074-7.436 | 0.800 |  |  |  |
| glycated hemoglobin | 0.824 | 0.174-3.903 | 0.087 |  |  |  |

**p* <0.05

^1^: In the multivariate analysis, positive indicators from the univariate analysis, as well as gender and age, were included as covariates.

Supplementary Table S2 Sensitivity analysis of mortality predictors

| Risk factor | Univariate analysis | | | Multivariate analysis^2^ | | |
| --- | --- | --- | --- | --- | --- | --- |
|  | OR | 95% CI | *P* value | OR | 95% CI | *P* value |
| Severe blood lactate elevation | 5.714 | 1.086-30.071 | 0.040* | 7.279 | 1.102-48.086 | 0.039* |
| Hemoglobin | 0.175 | 0.033-0.921 | 0.040* | 0.137 | 0.021-0.908 | 0.039* |

**p* <0.05

^2^: In the multivariate analysis, positive indicators from the univariate analysis, as well as gender ,age and follow-up duration were included as covariates.
